# Acute and 4-Week Repeated-Dose Oral Toxicity Studies of *Cirsium setidens* in Rats

**DOI:** 10.3390/molecules19067138

**Published:** 2014-05-30

**Authors:** Jong Seok Lee, Young-Hyun Kim, Dan-Bi Kim, Woo-Suk Bang, Ok-Hwan Lee

**Affiliations:** 1 Department of Food Science and Biotechnology, Kangwon National University, Chuncheon 200-701, Korea; E-Mails: jongseoklee78@gmail.com (J.S.L.); vvkyh@naver.com (Y.-H.K.); danbekim22@nate.com (D.-B.K.); 2 Department of Food and Nutrition, Yeungnam University, Gyeongsan, Gyeongbuk 712-749, Korea

**Keywords:** *Cirsium setidens*, oral administration, safety, toxicity

## Abstract

*Cirsium setidens* is a wild perennial plant species found in Korea that may have anti-oxidative, anti-adipogenic, and hepatoprotective activities. However, details of the toxicology of *C. setidens* remain unknown. This study was performed to evaluate the toxicological effects of an acute administration and 4-week repeated dosing of a *C. setidens* extract in Sprague-Dawley rats to ensure the safe use of this extract. *C. setidens* (1250, 2500, and 5000 mg/kg body weight/day) did not induce significant toxicological changes in groups matched by gender with respect to mortality, clinical signs, body weight, urinalysis, ophthalmoscopy, necropsy findings, hematology, and histopathology. Therefore, this study demonstrates that acute administration and 4-week repeated dosing of *C. setidens* extract orally using this administration protocol is safe.

## 1. Introduction

Plants have the ability to synthesize a wide variety of chemical compounds with important biological effects [1]. Many of these plant-derived compounds have beneficial effects on long-term health when consumed by humans and are commonly employed in developing countries for the treatment of various human diseases [2]. Many studies have shown that plant-derived compounds have a number of health benefits [3,4,5,6,7]. Epigallocatechin gallate has been extensively studied for insight into its beneficial health effects as a nutriceutical agent [8,9]. Morin hydrate, a member of flavonoid family, is shown to be an effective hepatoprotector *in vitro* and *in vivo* via modulation oxidative stress [10,11]. In particular, bioactive ingredients of *C. setidens* may exhibit hepatoprotective activity mainly via an SOD antioxidant mechanism [12]. However, herbs are composed of numerous bioactive chemicals, some of which may be toxic, and a number of studies have reported the toxic effects of herbal medicines [13].

*Cirsium setidens*, a wild perennial herb, has been used in Korean medicine for treating hemostasis, hematemesis, hematuria and hypertension [14]. Previous studies have demonstrated that *C. setidens* contains several bioactive ingredients with various medicinal activities, including cytotoxicity against human cancer cell lines [15,16]. Some studies have also reported that *C. setidens* shows hepatoprotective activity against CCl_4_- or D-galactosamine-induced liver damage via an antioxidant mechanism [12,17]. In a recent study, *C. setidens* inhibited hepatic fat accumulation by up-regulating the expression of fatty acid β-oxidation genes [18]. In our previous study, we found that *C. setidens* had an anti-adipogenic effect during the adipogenesis of 3T3-L1 cells [19]. However, despite several studies on the biological activity of *C. setidens*, there has been no toxicological study of *C. setidens*, to our knowledge.

Therefore, a systemic safety evaluation on the extract of *C. setidens* is necessary for the development of new foods or drugs. In this study, an aqueous extract from the dried *C. setidens* was prepared and its safety was evaluated in Sprague-Dawley (SD) rats by using an oral acute toxicity and a 4-week repeated-dose oral toxicity study, in compliance with the guidelines for testing the toxicity of pharmaceuticals from the Korea Food and Drug Administration (KFDA), using the Good Laboratory Practice regulations for Nonclinical Laboratory Studies.

## 2. Results and Discussion

### 2.1. Acute Oral Toxicity

Various bioactive compounds in herbal plants possess the capacity to significantly modulate the complex mechanisms involved in the pathology of chronic diseases such as cardiovascular disease, cancer, aging, and diabetes [20,21]. In addition, some ingredients have been reported to help improve psychiatric disorders such as depression [22,23,24]. In the search for new sources of health-promoting constituents in herbal plants, we focused on the *C. setidens* extract, which has recently shown hepatoprotective activities against CCl_4_- or D-galactosamine-induced liver damage and an anti-adipogenic effect during adipogenesis of 3T3-L1 cells via an antioxidant mechanism [12,17,19]. However, safety data are needed to assess potential toxicological concerns regarding the *C. setidens* extract. We therefore evaluated, for the first time, the acute oral safety/toxicity of *C. setidens* (Table 1)*.* Thus, *C. setidens* was orally administered once at doses of 1,250, 2,500 and 5,000 mg/kg BW in SD rats. Mortality, clinical signs, and body weight changes were observed for 14 days after administration. In addition, necropsy was performed to examine any abnormalities in the main organs. This study complied with the test guidelines from the Korea Food and Drug Administration (KFDA). No animal deaths or abnormal clinical signs related to the administration of *C. setidens* were observed during the experimental period. In addition, there were no significant changes in body weight in the *C. setidens-*treated groups compared with the control group. In addition, no abnormalities related to the administration of *C. setidens* were found during necropsy. These results suggest that the *C. setidens* extract is essentially non-toxic after acute exposure. We conclude that the no-observed-adverse-effect-level (NOAEL) of *C. setidens* is 5000 mg/kg.

**Table 1:** Toxicity results from acute oral administration of *C. setidens* in male and female rats.

Group ^a^	Dose (mg/kg)	Sex	Body Weights (g)			Clinical Signs	No Gross Finding	Mortality (Dead/Total)
			Day 0	Day 7	Day 14			
**G1**	**0**	**M ^b^**	153.8 ± 9.8	242.8 ± 10.6	304.7 ± 13.7	N **^d^**	5	0% (0/5)
		**F ^c^**	123.7 ± 7.3	173.7 ± 12.0	197.7 ± 11.0	N	5	0% (0/5)
**G2**	**1250**	**M**	153.9 ± 4.5	238.5 ± 10.5	307.4 ± 18.8	N	5	0% (0/5)
		**F**	125.1 ± 5.1	173.5 ± 11.6	198.2 ± 11.4	N	5	0% (0/5)
**G3**	**2500**	**M**	155.3 ± 4.5	242.4 ± 16.7	297.8 ± 19.6	N	5	0% (0/5)
		**F**	123.8 ± 3.7	168.8 ± 5.3	191.2 ± 8.5	N	5	0% (0/5)
**G4**	**5000**	**M**	157.8 ± 8.0	237.6 ± 15.0	293.0 ± 18.2	N	5	0% (0/5)
		**F**	124.4 ± 4.7	170.8 ± 11.7	191.7 ± 13.7	N	5	0% (0/5)

**^a^** G1, Control; G2, Group 2 (1250 mg/kg body weight); G3, Group 3 (2500 mg/kg body weight); G4, Group 4 (5000 mg/kg body weight); **^b^** M: Male; **^c^** F: Female; **^d^** N: Normal.

### 2.2. Four-Week Repeated-Dose Oral Toxicity

#### 2.2.1. General Appearance, Body Weight, Food Intake and Water Consumption

**Figure 1:**
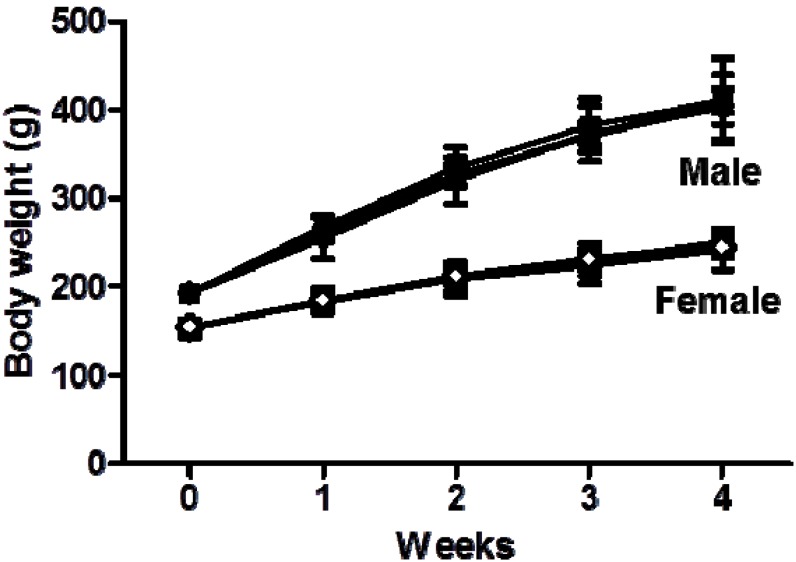
Growth curves for SD rats treated with *C. setidens* for 4 weeks. (● ○) Group 1 (control); (■ □) Group 2 (1250 mg/kg body weight per day); (▼ ▽) Group 3 (2500 mg/kg body weight per day); (◆ ◇) Group 4 (5000 mg/kg body weight per day).

Changes in general behavior and body weight are critical parameters for the objective evaluation of the effect of a compound on test animals because such changes are often the first signs of toxicity [25]. In a 4-week repeated-dose oral toxicity study, no animal deaths or abnormal clinical signs related to the administration of *C. setidens* were observed throughout the duration of the experiment at any dose used. The animals in the treatment groups did not appear seriously diseased at the end of the treatment period (data not shown). During the experimental period, a decrease in food consumption was observed in the 1250 and 2500 mg/kg female groups on week 4 (Table 2). As a result of water consumption, an increase was observed in the *C. setidens*-treated male groups administered 1,250 and 5000 mg/kg in week 1 and in the *C. setidens*-treated male groups in weeks 3 and 4 (all doses) compared to the control group (Table 3). In the female group, an increase in water consumption was observed in the group administered 5000 mg/kg in week 1 and in the groups administered 1250 and 5000 mg/kg in week 3 compared with the control group (Table 3). However, there were no significant changes in body weight in the *C. setidens-*treated groups compared with the control group throughout the study (Figure 1). Therefore, these changes were transient and appeared to be unrelated to the doses or to the treatment with *C. setidens*.

**Table 2:** Mean food consumption of male and female rats treated with *C. setidens* for 4 weeks.

Dose (mg/kg)	Mean Food Intake (g/rat/day) During Week			
	1	2	3	4
**Male**				
**0**	27.3 ± 0.2	27.8 ± 1.0	31.8 ± 0.4	31.0 ± 0.9
**1250**	26.9 ± 0.1	26.2 ± 0.2	31.6 ± 0.3	31.8 ± 0.1
**2500**	27.7 ± 0.7	26.3 ± 0.5	29.8 ± 2.8	30.8 ± 2.4
**5000**	27.5 ± 0.9	26.0 ± 0.4	30.0 ± 1.0	31.0 ± 1.1
**Female**				
**0**	19.8 ± 1.2	19.6 ± 1.6	20.8 ± 0.9	22.9 ± 3.0
**1250**	20.1 ± 0.5	17.4 ± 1.4	22.3 ± 0.7	18.6 ± 0.2 *
**2500**	19.5 ± 0.4	19.4 ± 1.1	20.8 ± 1.0	18.3 ± 0.0 *
**5000**	18.1 ± 0.3	18.2 ± 1.3	21.1 ± 0.9	21.1 ± 0.5

* Significant differences were compared with the control at *p* *<* 0.05.

**Table 3:** Mean water consumption of male and female rats treated with *C. setidens* for 4 weeks.

Dose (mg/kg)	Mean Water Consumption (g/rat/day) During Week			
	1	2	3	4
**Male**				
**0**	38.5 ± 2.2	40.7 ± 1.2	39.2 ± 2.3	34.9 ± 3.3
**1250**	43.5 ± 1.2 *	39.3 ± 6.4	47.6 ± 3.6 *	43.4 ± 2.2 *
**2500**	41.2 ± 3.2	38.9 ± 3.3	47.0 ± 1.9 *	48.5 ± 1.9 *
**5000**	45.7 ± 0.3 *	38.0 ± 2.0	58.6 ± 4.5 *	57.0 ± 0.2 *
**Female**				
**0**	29.9 ± 2.8	31.2 ± 4.9	27.1 ± 0.7	31.8 ± 1.3
**1250**	33.1 ± 1.8	26.2 ± 2.7	31.2 ± 1.8 *	27.0 ± 2.6
**2500**	31.7 ± 0.2	31.3 ± 1.4	32.8 ± 3.4	32.7 ± 2.4
**5000**	36.3 ± 0.9 *	33.0 ± 2.8	38.2 ± 1.4 *	32.5 ± 1.4

* Significant differences were compared with the control at *p* *<* 0.05.

#### 2.2.2. Urinary, Hematological and Biochemical Findings

In terms of urinary parameters such as specific gravity, pH, and the levels of leukocytes, nitrites, protein, glucose, ketones, blood, urobilinogen, and bilirubin, no treatment-related adverse effects were observed in male and female rats following the administration of *C. setidens* at doses of 1250, 2500, and 5000 mg/kg/day (Table 4). Bilirubin, a breakdown product of hemoglobin, is associated with hepatic diseases such as jaundice and ineffective erythropoiesis. Increased bilirubin levels reflect the extent of jaundice [26]. In the present study, no significant changes were observed in the levels of bilirubin in the *C. setidens*-treated and control rats, suggesting that *C. setidens* has no toxic effects on the erythropoietic system. In the 5000 mg/kg male group, although a high increase in protein excretion was noted, the other parameters exhibited no significant differences compared with the control group (Table 4).

Hematopoietic parameters are some of the most sensitive to assess the toxicity of drugs in humans and animals, and a blood profile usually gives vital information on the response of the body to injury or stress [27,28]. The hematological results for the male and female rats administered *C. setidens* are shown in Table 5. Hb concentration in female rats was increased at doses of 1,250 and 2,500 mg/kg *C. setidens.* Despite an increase in Hb concentration, this group of animals appeared normal, and the level stayed within the physiological range and appeared to be unrelated to the dose. This change was not observed in male *C. setidens*-treated rats. There were no consistent, statistically significant, dose-dependent adverse effects on any of the hematological parameters in the rats after 4 weeks of *C. setidens* treatment. In addition, there were no significant alterations in the biochemical parameters in all the groups treated with a 4-week repeated dose of *C. setidens* (Table 5). Symptoms of disorder in the liver and kidneys, which are the major internal organs in the body and have several important functions, appear only in serious disease [25]. The liver is the main site of the synthesis of plasma proteins, especially ALB. Changes in total serum proteins or the A/G ratio may indicate hepatic dysfunction [29]. In this study, the daily administration of *C. setidens* in feed for 4 weeks did not produce any significant changes in the TP, ALB, and A/G ratio of the treated rats from all the doses used compared with the control group (Table 5). The levels of serum biomarker enzymes such as ALP, AST, and ALT are usually assayed to evaluate any toxic effects on the liver [30]. In the present study, no significant increases in the levels of serum ALP, AST, and ALT in male and female rats were observed, although a few changes were noted (Table 5). These changes were not considered to be treatment related, as the changes were slight and the values were within the normal physiological range. CREA and BUN are known as important markers of kidney dysfunction [27]. No significant differences in CREA and BUN were found in treated rats compared with the control group. Changes in the BUN value were very slight and not dose-dependent (Table 5). These results suggest that *C. setidens* does not have a toxic effect on the liver and kidneys.

#### 2.2.3. Organ Weights and Histopathology

The absolute and relative organ weight data for males and females are summarized in Table 6. No significant treatment-related changes were observed in the absolute and relative organ weights of male and female rats following the administration of *C. setidens* at dosages of 1250, 2500, and 5000 mg/kg/day for 4 weeks.

**Table 4:** Urinalysis findings of male and female rats treated with *C. setidens* for 4 weeks.

Sex	Dose (mg/kg)	No. of Animals	Urinary Analysis on Week 4																			
			SG ^a^	Glu ^b^	Bili ^c^	Ketone ^d^			Blood ^e^	pH					Protein ^f^			Urobilinogen		Nitrite ^g^	Leukocyte ^h^	
				−	−	−	T	1+	−	6.5	7.0	7.5	8.0	8.5	−	T	1+	0.2	1.0	−	−	T
Male	**0**	5	1.014 ± 0.002	5	5	1	4	0	5	0	0	0	1	4	4	1	0	5	0	5	5	0
	**1250**	5	1.014 ± 0.002	5	5	5	0	0	5	0	0	0	0	5	5	0	0	5	0	5	5	0
	**2500**	5	1.010 ± 0.000	5	5	5	0	0	5	0	0	0	0	5	5	0	0	5	0	5	5	0
	**5000**	5	1.010 ± 0.000	5	5	3	1	1	5	0	0	0	0	5	0	3	2	5	0	5	5	0
Female	**0**	5	1.014 ± 0.002	5	5	5	0	0	5	0	0	0	0	5	0	5	0	2	3	5	5	0
	**1250**	5	1.010 ± 0.000	5	5	5	0	0	5	0	0	0	2	3	3	2	0	5	0	5	5	0
	**2500**	5	1.010 ± 0.000	5	5	5	0	0	5	0	0	0	0	5	2	3	0	5	0	5	5	0
	**5000**	5	1.011 ± 0.002	5	5	4	1	0	5	0	0	0	0	5	1	3	1	5	0	5	5	0

**^a^** SG: specific gravity; **^b^** Glucose, −: negative; **^c^** Bilirubin, −: negative; **^d^** Ketone, −: negative; T: 5 mg/dL; 1+: 15 mg/dL; **^e^** Blood, −: negative; **^f^** Protein, −: negative; T: <30 mg/dL; 1+: 30 mg/dL; **^g^** Nitrite: −: negative; **^h^** Leukocyte: −: negative; T: 15 Leu/μL.

**Table 5:** Hematological results and serum biochemical data of male and female SD rats treated orally with *C. setidens* for 4 weeks.

Sex	Male				Female			
Dose (mg/kg)	0	1250	2500	5000	0	1250	2500	5000
**WBC (K/μL)**	5.9 ± 1.1	7.1 ± 1.6	6.3 ± 1.4	5.9 ± 1.0	3.4 ± 0.1	5.1 ± 0.6	5.0 ± 0.9	5.2 ± 2.1
**RBC (M/μL)**	7.2 ± 0.3	7.2 ± 0.4	7.0 ± 0.2	7.0 ± 0.2	7.0 ± 0.1	7.7 ± 0.4	7.2 ± 0.2	7.1 ± 0.1
**Hb (g/dL)**	14.6 ± 0.4	14.4 ± 0.7	14.4 ± 0.4	14.2 ± 0.3	13.9 ± 0.5	15.3 ± 0.4	14.7 ± 0.3	14.4 ± 0.1
**PLT (K/μL)**	1319 ± 136	1374 ± 74	1369 ± 79	1391 ± 131	1364 ± 102	1340 ± 90	1342 ± 109	1251 ± 90
**PT (s)**	12.1 ± 2.0	11.0 ± 0.4	10.6 ± 1.3	9.6 ± 0.9	8.9 ± 0.8	9.8 ± 0.7	9.6 ± 2.2	8.8 ± 1.1
**APTT (s)**	20.7 ± 1.3	19.1 ± 2.2	19.8 ± 2.4	17.6 ± 1.6	18.2 ± 1.7	20.6 ± 4.2	17.9 ± 1.9	15.7 ± 1.4
**TP (g/dL)**	5.3 ± 0.2	5.5 ± 0.3	5.3 ± 0.3	5.5 ± 0.2	5.8 ± 0.5	5.7 ± 0.5	5.7 ± 0.3	5.6 ± 0.1
**ALB (g/dL)**	2.2 ± 0.1	2.3 ± 0.1	2.3 ± 0.1	2.3 ± 0.1	2.5 ± 0.2	2.5 ± 0.2	2.6 ± 0.1	2.6 ± 0.1
**T-BIL (mg/dL)**	0.02 ± 0.03	0.02 ± 0.03	0.00 ± 0.01	0.00 ± 0.00	0.00 ± 0.00	0.02 ± 0.02	0.02 ± 0.01	0.02 ± 0.01
**ALP (U/L)**	588 ± 138	644 ± 174	616 ± 96	587 ± 147	478 ± 74	450 ± 111	437 ± 159	421 ± 134
**AST (U/L)**	154 ± 22	154 ± 43	149 ± 27	146 ± 27	126 ± 39	136 ± 30	108 ± 31	125 ± 22
**ALT (U/L)**	29 ± 4	33 ± 6	34 ± 7	32 ± 6	24 ± 1	30 ± 8	24 ± 2	25 ± 4
**CREA (mg/dL)**	0.5 ± 0.0	0.5 ± 0.0	0.5 ± 0.0	0.5 ± 0.0	0.5 ± 0.1	0.5 ± 0.1	0.5±0.1	0.6 ± 0.0
**BUN (mg/dL)**	12.2 ± 0.6	12.0 ± 1.8	14.1 ± 0.6	13.5 ± 0.9	15.2 ± 1.4	18.0 ± 1.2	17.3 ± 1.6	17.5 ± 1.6
**TC (mg/dL)**	56 ± 13	64 ± 13	56 ± 10	55 ± 12	85 ± 16	66 ± 10	76 ± 6	71 ± 6
**TG (mg/dL)**	85 ± 15	115 ± 51	77 ± 44	81 ± 30	31 ± 11	18 ± 8	32 ± 8	18 ± 4
**GLU (mg/dL)**	116 ± 13	140 ± 42	131 ± 30	92 ± 13	126 ± 25	110 ± 12	111 ± 13	92 ± 13
**Ca (mg/dL)**	9.9 ± 0.3	10.0 ± 0.3	9.9 ± 0.2	10.2 ± 0.2	10.0 ± 0.2	10.2 ± 0.5	10.3 ± 0.2	10.2 ± 0.3
**Na (mmol/L)**	141.3 ± 1.1	140.4 ± 0.6	139.4 ± 0.9	139.6 ± 1.0	140.0 ± 0.8	140.0 ± 0.8	139.3 ± 0.5	138.0 ± 0.4
**K (mmol/L)**	4.5 ± 0.3	4.4 ± 0.2	4.8 ± 0.2	4.7 ± 0.2	4.2 ± 0.2	4.3 ± 0.4	4.3 ± 0.2	4.6 ± 0.5
**Cl (mmol/L)**	107.5 ± 2.0	105.4 ± 1.5	105.8 ± 1.2	105.5 ± 0.8	108.0 ± 1.6	106.4 ± 1.9	104.8 ± 1.0	104.8 ± 0.5

**Table 6:** Absolute and relative organ weights of male and female rats treated orally with *C. setidens* for 4 weeks.

Sex	Male				Female			
Dose (mg/kg)	0	1250	2500	5000	0	1250	2500	5000
**Absolute Body Weight**								
**Liver (g)**	11.05 ± 1.60	11.04 ± 0.99	11.39 ± 1.60	11.34 ± 0.58	6.66 ± 0.46	6.15 ± 0.58	6.70 ± 0.52	6.65 ± 0.75
**Kidneys (g)**	3.01 ± 0.31	2.97 ± 0.18	3.01 ± 0.32	2.94 ± 0.13	1.82 ± 0.11	1.78 ± 0.09	1.92 ± 0.16	1.84 ± 0.17
**Spleen (g)**	0.85 ± 0.09	0.88 ± 0.32	0.80 ± 0.09	0.82 ± 0.11	0.53 ± 0.08	0.52 ± 0.09	0.52 ± 0.09	0.53 ± 0.10
**Lungs (g)**	1.71 ± 0.41	1.61 ± 0.08	1.64 ± 0.11	1.64 ± 0.15	1.25 ± 0.04	1.19 ± 0.09	1.25 ± 0.09	1.15 ± 0.08
**Heart (g)**	1.49 ± 0.18	1.50 ± 0.12	1.5 1± 0.16	1.60 ± 0.20	1.01 ± 0.10	0.96 ± 0.18	0.98 ± 0.07	0.93 ± 0.05
**Relative Body Weight**								
**Body Weight (g)**	379.4 ± 32.4	379.4 ± 14.1	386.9 ± 37.7	378.4 ± 18.0	231.7 ± 12.5	226.4 ± 4.3	220.8 ± 23.6	226.6 ± 12.7
**Liver (%)**	2.90 ± 0.20	2.91 ± 0.19	2.94 ± 0.21	3.00 ± 0.08	2.88 ± 0.21	2.72 ± 0.29	3.05 ± 0.29	2.93 ± 0.24
**Kidneys (%)**	0.40 ± 0.02	0.40 ± 0.02	0.40 ± 0.03	0.39 ± 0.02	0.39 ± 0.04	0.39 ± 0.02	0.43 ± 0.05	0.40 ± 0.03
**Spleen (%)**	0.23 ± 0.03	0.23 ± 0.09	0.21 ± 0.03	0.22 ± 0.02	0.23 ± 0.03	0.23 ± 0.04	0.24 ± 0.02	0.23 ± 0.05
**Lungs (%)**	0.46 ± 0.13	0.43 ± 0.03	0.43 ± 0.04	0.43 ± 0.03	0.54 ± 0.02	0.52 ± 0.05	0.57 ± 0.04	0.51 ± 0.02
**Heart (%)**	0.39 ± 0.06	0.40 ± 0.04	0.39 ± 0.04	0.42 ± 0.04	0.44 ± 0.04	0.42 ± 0.08	0.44 ± 0.05	0.41 ± 0.03

Gross examination of internal organs, including the brain, pituitary gland, thymus, lungs, heart, spleen, liver, adrenals, kidneys, thyroids, testis (males), prostate gland (males), uterus (females), and ovaries (females), of the control and *C. setidens*-treated rats and histopathological examination in the main organs (liver, spleen, heart, kidneys, and lungs) did not show any abnormal findings (data not shown). Therefore, these results indicate that the oral administration of *C. setidens* for 4 weeks did not show toxic effects in rats.

## 3. Experimental Section

### 3.1. Animal Husbandry and Maintenance

A total of 80 specific pathogen-free, 6-week-old SD rats, which included 40 males weighing between 139.8 g and 201.9 g and 40 females weighing between 115.6 g and 167.7 g, were purchased from Orient Bio Co. (Kapyong, Korea) and used after a week of quarantine and acclimatization. The animal facility is maintained at 22 ± 3 °C, relative humidity of 50% ± 20%, air ventilation of 10-15 times/h, and a 12 h light/dark cycle (light 08:00-20:00) at 150-300 Lux (Korea Testing and Research Institute, Seoul, Korea). The animals were fed sterilized tap water and commercial rodent chow (Cargill Co. Gunsan, Korea) *ad libitum*. This experiment was conducted in facilities approved by the Association for Assessment and Accreditation of Laboratory Animal Care (AAALAC) International. All procedures were approved by the Institutional Animal Care and Use Committee (IACUC).

### 3.2. Test Substance

*C. setidens* (10 kg) was extracted with distilled water (200 L) at 90 °C for 4 h and then allowed to cool at room temperature. The extractive solution was ﬁltered with Whatman ﬁlter paper (0.2 µm), concentrated to 15 brix using a vacuum evaporator (Eyela, Tokyo, Japan) at 80 °C, and then freeze-dried for 96 h using a freeze dryer (Ilshin, Seoul, Korea). The extract of *C. setidens* was dissolved in distilled water for oral administration. The phenolic compounds in the samples were analyzed using an HPLC-DAD analyses were performed on an Agilent series 1100 HPLC instrument allowing the determination at different wavelengths (280, 320, and 370 nm) of single molecules belonging to different subclasses [31]. In detail, the Nucleosil 100-5 C-18 column (Macherey-Nagel, Diiren, Germany; 250 mm × 4.0 mm i.d., 5 μm particle size) which was protected by a 10 mm guard column was used. The eluents were water at pH 3.29 by formic acid (0.035%, *v*/*v*) (A) and acetonitrile (B). The analyses were performed by a multistep linear solvent gradient as follows: 0%-15% B (45 min): 15%-30% B (15 min): 30%-50% B (5 min), 50%-100% B (5 min) and 100%-0% B (10 min). The diode array detector monitored at 270 nm, and the injection volume of the samples was 10 µL.

The standards used for the analysis were 4-hydroxyl benzohydrazide, gallic acid, vanillic acid, *p*-anisic acid, alizarin, chlorogenic acid, caffeic acid, syringic acid, *p*-coumaric acid, trans-ferulic acid, catechin, epigallocatechin gallate, quercetin hydrate, myricetin, morin hydrate, 3-hydroxyflavone, rutin, and naringin which were purchase from Sigma (St. Louis, MO, USA). The results are shown in Table 7.

**Table 7:** The major phytoconstituents composition of aqueous extracts of *C. setidens*.

Compounds	Contents (mg/100 g Extract)
*Phenolic acid*	
Phloroglucinol	ND
4-Hydroxybenzhydrazide derivative	72.77
Gallic acid	15.39
Vanillic acid	48.90
Protocatechuic acid ethyl ester	19.98
2-Amino-3,4-dimethylbenzoic acid	ND
*p*-Anisic acid	23.24
Chlorogenic acid	53.04
Caffeic acid	36.30
Syringic acid	0.87
*p*-Coumaric acid	8.20
Chlorogenic derivative	ND
*trans*-Ferulic acid	18.24
**Total of phenolic acid**	**296.93**
***Flavonoids***	
(+)-Catechin hydrate	93.04
Gallocatechin	264.05
(−)-Epigallocatechin	2376.00
Epicatechin	18.96
Epigallocatechin gallate	52.25
Quercetin hydrate	20.31
Myricetin	167.69
Morin hydrate	610.07
Quercetin dihydrate	8.81
3-Hydroxyflavone	13.07
Rutin hydrate	63.14
Naringin	74.51
**Total of flavonoids**	**3761.90**

ND, not detected.

### 3.3. Acute Oral Toxicity

*C. setidens* (0, 1250, 2500, and 5000 mg/kg) was administered by oral gavage to age-matched male and female SD rats. Forty SD rats were randomly divided into a control (Group 1, *n* = 10 [5 males and 5 females]) and three dosages (Groups 2, 3, and 4 with 1250, 2500, and 5000 mg/kg/day, respectively) with equal numbers of males and females. Samples were administered once by oral gavage at 10 mL/kg of body weight (BW). The control animals were treated with the same volume of distilled water. Rats were anesthetized with diethyl ether followed by cervical decapitation. During the experimental period, all animals were observed one or more times per day. However, after administration, all animals were observed every half hour for 4 h. Observation was performed for 14 days post-administration. Body weights were measured at the initiation of treatment, and 7 and 14 days post-administration. On day 14 after administration, the animals were sacrificed and the external surface, thoracic organs, abdominal organs and their contents were macroscopically examined.

### 3.4. Four-Week Repeated-Dose Oral Toxicity

#### 3.4.1. Study Design

*C. setidens* (0, 1250, 2500, and 5000 mg/kg) was administered by oral gavage to male and female SD rats. Forty SD rats were randomly divided into a control (Group 1, *n* = 10 [five males and five females]) and three dosages (Groups 2, 3, and 4 with 1250, 2500, and 5000 mg/kg/day, respectively) with equal numbers of males and females. Samples were administered by oral gavage at 10 mL/kg of BW on a daily basis 7 days a week for 4 weeks. The control animals were treated with the same volume of distilled water. During the administration period, the animals were observed for general appearance twice daily, and body weight, food intake, and water consumption were recorded once a week.

#### 3.4.2. Urinalysis

On the last week of the administration period, a urine test was performed with fresh urine to determine specific gravity, pH, and levels of leukocytes, nitrites, protein, glucose, ketones, blood, urobilinogen, and bilirubin using a Clinitek 500 urine chemistry analyzer (Bayer Health Care, Cambridge, MA, USA) and a Multistix 10SG (Bayer, IN, USA). The microscopic examination of urinary sediments and urinary color was conducted with fresh urine from the control and highest dose groups after collecting for 3 h. The urine volume was checked after collecting for 24 h.

#### 3.4.3. Ophthalmoscopy

External eye examinations were performed shortly before the start of the experiment and before the termination of treatment. The ocular fundus was examined before the termination of treatment using an indirect binocular ophthalmoscope on the negative control group and the highest dose group.

#### 3.4.4. Hematology and Serum Biochemistry

Hematological and biochemical studies of liver function, plasma constituents, and electrolyte concentrations were determined using standard clinical procedures. The animals were fasted overnight prior to necropsy and blood collection. Blood samples were taken from the abdominal aorta using a syringe under isoflurane anesthesia. Blood samples were collected into CBC bottles containing EDTA-3K (Sewon Medical, Seoul, Korea) and analyzed within 20 min. Hematological parameters were determined using an ADVIA 120 hematology analyzer (Siemens Medical Solutions Diagnostics, Tarrytown, NY, USA), including total leucocyte count (WBC), red blood cell (RBC) count, hemoglobin (Hb) concentration, platelet (PLT) count, prothrombin time (PT), and activated partial thrombo-plastin time (APTT).

Serum biochemical parameters, measured with a Daiichi diagnostics reagent kit and an automated serum analyzer (Hitachi 7060 chemistry analyzer, Tokyo, Japan) and electolyte analyzer (Bayer 644 Na/K/Cl analyzer, PA, USA), were total serum protein (TP), albumin (ALB), total bilirubin (T-BIL), alkaline phosphatase (ALP), aspartate aminotransferase (AST), alanine aminotransferase (ALT), creatinine (CREA), blood urea nitrogen (BUN), total cholesterol (TC), triglycerides (TG), glucose (GLU), and electrolytes such as calcium (Ca), sodium (Na), potassium (K), and chloride (Cl).

#### 3.4.5. Necropsy

At the end of the treatment period, all male and female SD rats were anesthetized by isoflurane and then sacrificed by exsanguination from the carotid artery. Complete gross postmortem examinations were performed on all animals. All organs were carefully examined macroscopically and the brain, pituitary gland, thymus, lungs, heart, spleen, liver, adrenals, kidneys, thyroids, testis (males), prostate gland (males), uterus (females), and ovaries (females), were weighed relative to the total body weight.

#### 3.4.6. Histopathology

Organs were fixed and preserved in 10% phosphate-buffered formalin. Fixed tissues were routinely processed for embedding in paraffin, sectioned, and stained with hematoxylin and eosin. Collected tissues were grossly and microscopically examined.

### 3.5. Statistical Analysis

Data are expressed as the means ± SEM and analyzed by a one-way analysis of variance (ANOVA) followed by Fisher’s Least Significant Difference test using SPSS software version 10 [32]. *p* *<* 0.05 was considered a significant difference as compared with control.

## 4. Conclusions

In summary, our toxicological studies revealed no toxic effects of oral administration of *C. setidens* during acute administration and 4-week repeated doses in male or female rats. A chronic toxicity study should be further performed to assess the long-term safety of the *C. setidens* extract.
